# Diversity in the Glucose Transporter-4 Gene (*SLC2A4*) in Humans Reflects the Action of Natural Selection along the Old-World Primates Evolution

**DOI:** 10.1371/journal.pone.0009827

**Published:** 2010-03-23

**Authors:** Eduardo Tarazona-Santos, Cristina Fabbri, Meredith Yeager, Wagner C. Magalhaes, Laurie Burdett, Andrew Crenshaw, Davide Pettener, Stephen J. Chanock

**Affiliations:** 1 Laboratory of Translational Genomics, Division of Cancer Epidemiology and Genetics, National Cancer Institute, National Institutes of Health, Bethesda, Maryland, United States of America; 2 Departamento de Biologia Geral, Instituto de Ciências Biológicas, Universidade Federal de Minas Gerais, Belo Horizonte, Minas Gerais, Brazil; 3 Dipartimento di Biologia Evoluzionistica Sperimentale, Università di Bologna, Bologna, Italy; 4 Intramural Research Support Program, SAIC Frederick, National Cancer Institute - Frederick Cancer Research and Development Center (NCI-FCRDC), Frederick, Maryland, United States of America; 5 Core Genotype Facility, National Cancer Institute, National Institutes of Health, Gaithersburg, Maryland, United States of America; Innsbruck Medical University, Austria

## Abstract

**Background:**

Glucose is an important source of energy for living organisms. In vertebrates it is ingested with the diet and transported into the cells by conserved mechanisms and molecules, such as the trans-membrane Glucose Transporters (GLUTs). Members of this family have tissue specific expression, biochemical properties and physiologic functions that together regulate glucose levels and distribution. GLUT4 –coded by *SLC2A4* (17p13) is an insulin-sensitive transporter with a critical role in glucose homeostasis and diabetes pathogenesis, preferentially expressed in the adipose tissue, heart muscle and skeletal muscle. We tested the hypothesis that natural selection acted on *SLC2A4*.

**Methodology/Principal Findings:**

We re-sequenced *SLC2A4* and genotyped 104 SNPs along a ∼1 Mb region flanking this gene in 102 ethnically diverse individuals. Across the studied populations (African, European, Asian and Latin-American), all the eight common SNPs are concentrated in the N-terminal region upstream of exon 7 (∼3700 bp), while the C-terminal region downstream of intron 6 (∼2600 bp) harbors only 6 singletons, a pattern that is not compatible with neutrality for this part of the gene. Tests of neutrality based on comparative genomics suggest that: (1) episodes of natural selection (likely a selective sweep) predating the coalescent of human lineages, within the last 25 million years, account for the observed reduced diversity downstream of intron 6 and, (2) the target of natural selection may not be in the *SLC2A4* coding sequence.

**Conclusions:**

We propose that the contrast in the pattern of genetic variation between the N-terminal and C-terminal regions are signatures of the action of natural selection and thus follow-up studies should investigate the functional importance of differnet regions of the *SLC2A4* gene.

## Introduction

Glucose is an important source of energy for living organisms. In vertebrates, it can be ingested with the diet and transported into the cells by conserved mechanisms and molecules, such as the trans-membrane Glucose Transporters (GLUTs) protein family. Members of this family have tissue specific expression, biochemical properties and physiologic functions that together, contribute to the regulation of blood sugar levels as well as its distribution. GLUT4 –coded by *SLC2A4* (chromosome 17p13), is an insulin-sensitive glucose transporter with a critical role in glucose homeostasis. In absence of insulin, GLUT4 is maintained sequestered in intracellular vesicles in tissues where it is preferentially expressed: adipose tissue, heart muscle and skeletal muscle [1], [2]. Within minutes of insulin stimulation, GLUT4 molecules move to the cell surface to transport glucose into the cell, reducing blood glucose and allowing the intracellular synthesis of glycogen and triglycerides. GLUT4 also plays a role during prolonged exercise [3], when demand for glucose by contracting muscles is associated with its translocation from intracellular vesicles to the cell membrane to favor glucose uptake. Based on the critical role of GLUT4 in glucose homeostasis, and the association of hyperglycemia with metabolic disorders such as insulin resistance, type-2 diabetes, dyslipidaemia, hypertension and obesity [4], [5], structural and functional studies of GLUT4 have received great attention: a Pubmed search using the query “GLUT4 and glucose transporter” reports 250 publications in 2008 and 940 during the 2004–2008 quinquennium. On a structural basis, the GLUT4 protein has 12 membrane-spanning domains, with both the amino and carboxyl termini intracellularly oriented. Moreover, the human GLUT4 promoter region has been identified within 895 bp upstream of the transcription initiation site, containing *cis* regulatory domains for the Myocyte Enhancer Factor 2 and the Domain I Binding Protein, both required for regulation of transcription [6].

Forty-six years ago, James Neel posited the “thrifty” genotype hypothesis, suggesting that variants that increase diabetes type II susceptibility under modern life were advantageous in past environments characterized by food shortage [7]. He noticed that in human populations, diabetic offspring tend to be weightier than non-diabetics offspring, and that “the diabetic genotype” was a “thrifty genotype, in the sense of being exceptionally efficient in the intake and/or utilization of food”. Recently, Anna Di Rienzo and colleagues have tested and discussed this hypothesis in a modern population genetics framework [8], [9] and have shown that, consistent with the Neel hypothesis, the pattern of diversity of *Calpain-10* (*CAPN10*), a candidate gene with polymorphisms associated with diabetes type II, suggests evidence of balancing natural selection. In this context, it is important to test if the diversity of other genes playing a role in glucose metabolism, such as *SLC2A4*, also bears the signature of natural selection. Moreover, because glucose metabolism is critical for energy availability across all living organisms, it is important to infer if a signature of natural selection is recent or if, alternatively, it predates the coalescent of human lineages. Indeed, genes involved in glucose metabolism are overrepresented among genes that have experienced positive selection in its promoter region during human evolution [10]. To address these issues, we re-sequenced the *SLC2A4* locus in 102 ethnically diverse individuals and described its pattern of diversity in different populations. We compared the pattern of human polymorphisms with divergence from other mammals and tested the hypothesis that natural selection has shaped *SLC2A4* diversity.

## Materials and Methods

### Samples

Two datasets of anonymous samples were used. The first one (i.e. the re-sequencing panel) was composed by 102 unrelated individuals of the SNP500Cancer project (http://snp500cancer.nci.nih.gov/) [11], which includes: 24 African ancestry (15 African Americans from the United States and 9 Pygmies), 23 admixed Latin American (from Mexico, Puerto Rico and South America), 31 Europeans (from the CEPH/UTAH pedigree and the NIEHS Environmental Genome Project) and 24 Asians-Oceanians (from Melanesia, Pakistan, China, Cambodia, Japan and Taiwan). The second dataset (i.e. the SNPs-panel) includes a subset of 280 individuals from the HGDP-CEPH Panel [12], belonging to the following 13 populations: (http://snp500cancer.nci.nih.gov/terms_ethnic_hdp.cfm): San, Bantu, Mandenka and Yoruba from Sub-Saharian Africa; Sindhi, Pathan and Han from Asia; French, North-Italian, Tuscan and Orcadian from Europe; and Pima and Maya from the Americas.

### PCR amplification, sequencing and SNPs genotyping

In the re-sequencing panel, we performed bi-directional sequencing of 6311 bp per individual, encompassing the most of the *SLC2A4* gene and ∼1 kb upstream the gene (Reference sequence: chromosome 17, positions 7124832-7131142 of the NCBI human genome build 36.3). A fragment of 949 bp at the end of the 3'UTR could not be reliably sequenced because of a high density of A/T bases. For PCR amplification and sequencing we followed the protocol described by Packer et al. [11]. The orthologous chimpanzee and rhesus genomic sequences were used to determine ancestral states of polymorphisms. For analysis of long range linkage disequilibrium, we used data from 56 and 48 SNPs mapped ∼0.5 Mb upstream and downstream of *SLC2A4* from the *Affymetrix SNP Array 5.0*, genotyped in the SNP500Cancer individuals ([13], see supplementary File S1 for the list of SNPs).

In the SNPs-panel we genotyped 5 common and representative *SLC2A4* SNPs (i.e. tag-SNPs *in sensu* Carlson et al. [14], see below for the criteria used for tag-SNPs selection) identified in the re-sequencing panel: rs5418, rs16956647, rs5435, rs5436, and rs5417. For this genotyping, we used Taqman assays (Applied Biosystems, Foster City, CA, US) following the protocols described in http://snp500cancer.nci.nih.gov/.

### Evolutionary and population genetics analyses

We tested the Hardy-Weinberg equilibrium using the test of Guo and Thompson [15], implemented in the software Arlequin 3.0 [16]. Insertion-deletions (INDELs) were excluded from further population genetics analyses. We assessed intra-population variability in the following way: For the re-sequencing data we used estimators of the θ parameter based on the infinite-site-model of mutations: π, the per-site mean number of pair-wise differences between sequences [17], and by θw, based on the number of segregating sites (S) [18]. Instead, for the SNPs-panel, we calculated from haplotyes the gene diversity *in sensu* Nei et al. [19]. We measured pair-wise between-populations diversity measuring its percentage of the total genetic variance present in both populations (F_ST_), and we also performed the Analysis of Molecular Variance (AMOVA) to measure the apportionment of genetic variance within and among populations or groups of populations [20], using the software Arlequin 3.0.

We inferred haplotypes considering SNPs with a Minor Allele Frequency (MAF) ≥0.05 in at least one population, using the method by Stephens and Sheet [21], that takes into account decay of linkage disequilibrium with distance among SNPs. The recombination parameter ρ was also calculated for each population from the re-sequencing panel by using the method of Li and Stephens [22]. These inferences were performed by the software Phase v.2.1.1 (see supplementary File S1 for additional specifications). Graphical relationships between haplotypes of the re-sequencing panel were explored by a Reduced Median Network, as implemented in the software Network 4.1.1.2 [23].

To investigate if the observed patterns of variability in human population is consistent with the neutral model, we used the tests of Tajima's D [24], Fu and Li's D* and Fu and Li 's F* [25] on the re-sequencing panel. In addition to the standard null hypothesis of neutrality under constant population size, we tested for the African population the significance of these statistics against a family of null hypotheses that consider scenarios of exponential demographic growth, which is consistent with its demographic history, in particular since the Pleistocene-Holocene [26]. We constructed the distribution of the statistics to be tested under these null hypotheses using the software ms [27] (see supplementary File S1 for details).

Linkage disequilibrium (LD) was estimated by r^2^ [28] for SNPs with MAF≥0.05 in at least one population and its significance assessed by LOD scores, using software Haploview v.3.2 [29], [30]. Based on the pattern of intragenic LD that emerged from the re-sequencing panel, we identified *SLC2A4* multi-population tag-SNPs (that may be used as surrogates for untyped SNPs [13]), with a threshold r^2^>0.64. For analyses of long range LD using the 104 *Affymetrix* SNPs covering ∼1 Mb region, we first inferred long-range haplotypes using the algorithm by Scheet and Stephens [31], implemented in the software fastPHASE.v130.beta (details in supplementary File S1). We tested for the presence of recombination hotspots along the ∼1 Mb using the approximate marginal likelihood method by Fearnhead [32] implemented in the software SequenceLDhot. For the long-range phased data, we applied the test for positive natural selection of Sabeti et al. [33], based on the Extended-Haplotype-Homozygosity statistic, which measures if a specific allele/haplotype under selection shows a higher LD with the sorrounding genomic region. We applied this test using haplotypes of the 8 common *SLC2A4* SNPs. Data handling for population genetics analyses were perfomed using a set of scripts from the platform DIVERGENOME (developed by Magalhães WCS and Tarazona-Santos ET).

To explore evolutionary conservation across different species, we measured for each polymorphic position the conservation score of the Genome Browser website (assembly March 2006, http://genome.ucsc.edu/), based on multiple alignment of 17 vertebrate species [34]. To test the fitness of the data to the neutral model including inter-specific comparisons, we performed neutrality tests based on the comparison of polymorphisms and divergence rates from chimpanzee and rhesus: the McDonald and Kreitman test [35] that compares synonymous (assumed to be neutral) and nonsynonymous sites; and the adaptation of the Kolmogorov-Smirnov statistic (D_KS_) by McDonald [36], developed to test the hypothesis that the ratio of polymorphisms to divergence is homogeneous along a genomic region. This statistic is based on the maximum absolute difference between the observed and expected cumulative numbers of polymorphisms. These tests were performed by DNAsp 4.10 and Slider softwares, respectively. To gain insights into the evolutionary history of *SLC2A4* at a larger evolutionary scale, we identified regions in the coding sequence associated to different kinds of selection through the evolutionary history of mammals. We compared *SLC2A4* coding sequences among the following mammals for which information is publicly available: *H. sapiens* (NM_001042.2), *P. troglodytes* (XM_001155036.1), *M. mulatta* (XM_001107391.1), *B. taurus* (NM_174604.1), *M. musculus* (NM_009204.2), *R. norvegicus* (NM_012751.1), *S. scrofa* (NM_001128433.1), *E. caballus* (NM_001081866.1). We used the maximum likelihood approach developed by Yang [37] to estimate ratios of non-synonymous (dN) to synonymous (dS) substitutions (ω  =  dN/dS) for *SLC2A4* codons under a variety of evolutionary models (see supplementary File S1). This method allows inferences about the evolution of a coding region along a phylogeny and to discriminate among codons that have evolved under strong or weak purifying selection, neutrality or adaptive positive selection. After fitting the data to an appropriate evolutionary model, a Bayes Empirical Bayes approach was used to infer the ω parameter for each codon. We performed this analysis using the software PAML [38].

## Results

**Figure 1:**
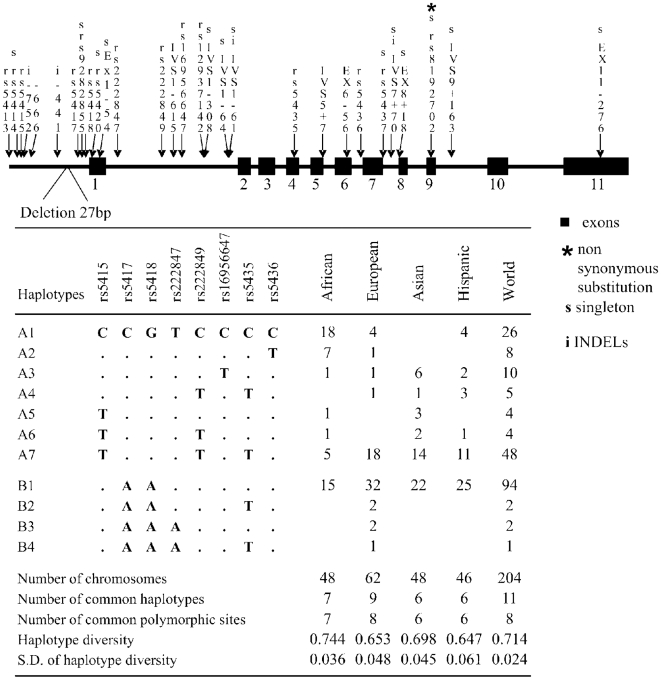
Genomic structure of SLC2A4, substitutions found, inferred haplotypes and their frequencies. Substitutions are represented by arrows and when no dbSNP name is available, named as in the SNP500Cancer database. A total of 29 polymorphisms (25 SNPs and 4 INDELs) were detected in the 204 worldwide re-sequenced chromosomes. Forty five percent of the substitutions were singletons and only 8 reached a MAF>0.05 in at least one studied population. Comparison with the homologous chimpanzee sequence suggests that for all SNPs the ancestral allele is modal in humans. In the human genome, there is a 27 bp fixed deletion 348 bp upstream of the transcription initiation site. Three non-coding SNPs are in evolutionarily conserved positions (UCSC Genome Browser, [33]): rs5415 (conservation score: 0.96), within the promoter region, as well as rs222847 and rs222849, both with conservation score of 0.99 and within the first intron. Only one of the 4 coding-SNPs is non-synonymous (rs8192702, Ala358Val, a conservative substitution in exon 9, in the ninth trans-membrane domain), observed in a European. Haplotypes are inferred using only the 8 common SNPs.

By re-sequencing the *SLC2A4* gene and ∼1 kb upstream it, we detected 29 polymorphisms, including one non-synonymous singleton in exon 9 (Figure 1). All SNPs/INDELs fit Hardy-Weinberg proportions in the studied populations, both in the re-sequenced and the follow-up SNP genotyping. Two features of the observed pattern of diversity are interesting. First, across the four studied populations, all the eight common SNPs are concentrated upstream of exon 7 (on the first ∼3700 bp of the gene), while the region downstream of intron 6 (∼2600 bp) only harbors 6 singletons in Europeans/Africans, and no variation in Asians and Latin Americans. This lack of common variation in the C-terminal part of the gene is even more surprising after verifying trough the UCSC Genome Browser that among mammals, the genomic region downstream of intron 6 is as much variable as the region upstream of exon 7 (data not shown). Second, the African set shows a larger Watterson's θ (which depends on the number of segregating sites), but unexpectedly, they show a lower nucleotide diversity (which mostly depends on common variants, π*_SLC2A4_* = 0.00038) than non-Africans (Table 1, [39], [40], [41]. For most of the human genome, African populations show larger π values than non-Africans, which is likely due to the bottleneck occurred approximately 40–50 thousand years ago during the migration of humans “Out of Africa” [42]. The observed π*_SLC2A4_* in the African population is also the twenty-second lowest value when compared with 329 re-sequenced genes (seventh percentil of the distribution, december 2009) analyzed in an African-American sample by the Seattle SNPs initiative (see http://pga.gs.washington.edu/summary_stats.html and [43]). Therefore, in addition to the lack of common variation downstream of intron 6 in humans, *SLC2A4* has an uncommon pattern of variation in Africans, characterized by a high number of segregating sites and singletons but low nucleotide diversity.

**Table 1:** Summary of intra-population diversity indexes and tests of neutrality based on re-sequencing analysis of the four SNP500Cancer populations.

Populations	African	European	Asian	Hispanic	World
N. of chromosomes	48	62	48	46	204
Segregating sites	20	13	8	9	25
Singletons	13	5	1	3	11
Common SNPs (MAF^a^>0.05)	6	6	6	5	5
ρ (per gene)	1.70	0.48	0.45	0.63	7.34
*θ estimators*					
π ± SD (×10^−3^)	0.38±0.04	0.43±0.03	0.44±0.02	0.40±0.04	0.43±0.02
θ_W_ ± SD (×10^−3^) (per site)	0.71±0.25	0.44±0.16	0.29±0.13	0.32±0.14	0.67±0.19
*Neutrality tests*					
Tajima's D	−1.483	−0.064	1.453	0.587	−1.016
Fu and Li's D^*^	−3.069 b	−1.176	0.594	−0.656	−2.986 b
Fu and Li's F^*^	−2.992 b	−0.941	1.023	−0.226	−2.630 c
P of McDonald-Kreitman test	0.544	1.000	1.000	1.000	1.000

a Minor Allele Frequency.

b P<0.02.

c P<0.05.

**Figure 2:**
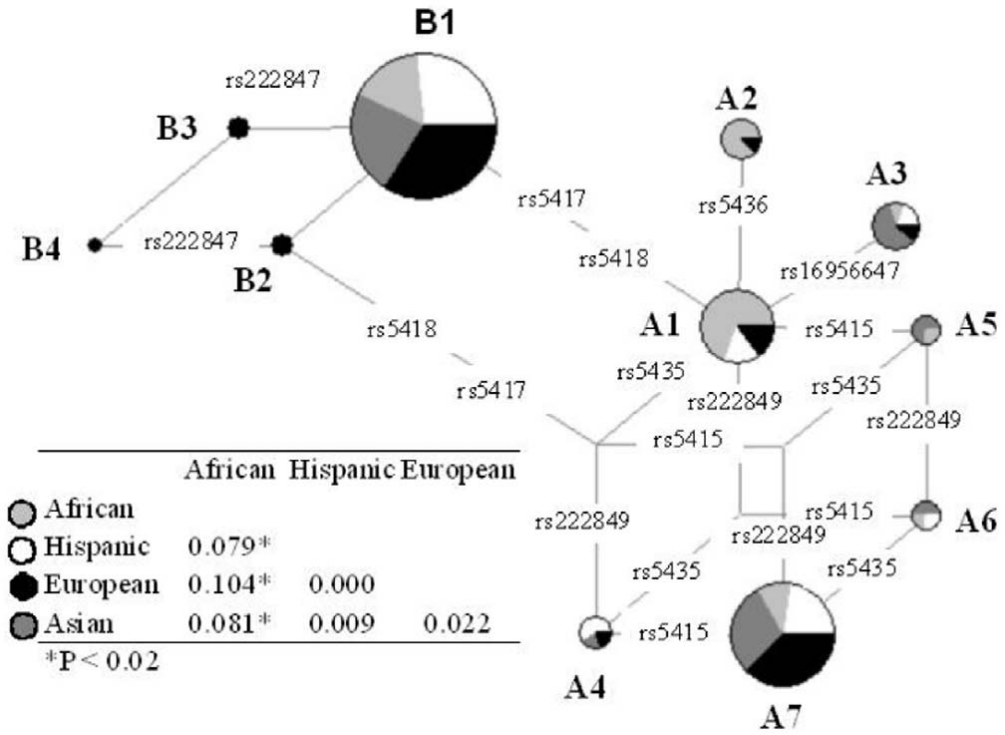
Reduced Median Network of SLC2A4 haplotypes inferred in the re-sequencing panel and matrix of pairwise F_ST_. Haplotypes were inferred from the 8 polymorphisms with a MAF <0.05 in at least one population. Each circle represents a different haplotype, its size is proportional to its relative frequency and the presence in each population is indicated with different gray tonalities. Base substitutions are indicated along branches. The reticulated network reflects the action of recombination or recurrent substitution.

Based on the 8 common polymorphisms with a MAF≥0.05 in at least one population (all located upstream of exon 7) we inferred 11 haplotypes (Figure 1). The Reduced Median Network in Figure 2 illustrates the phylogenetic relationships among haplotypes and their distribution in human populations. The differentiation between human populations (F_ST_) observed in the re-sequencing panel for *SLC2A4* is 3.8% (P = 0.013), which is lower than the 10–12% observed on average among human populations [44]. This result reflects the fact that only the African population is differentiated from the homogeneous non-African ones, which is mainly due to differences in frequencies of haplotypes A2 and A7 (Figure 2). The analysis of the SNPs-panel produced results that were consistent with those of the re-sequencing panel (see details see the supplementary File S1).

Based on the observed pattern of diversity of *SLC2A4*, we tested the hypothesis that it was shaped by natural selection. We interrogated the evolutionary basis of the low nucleotide diversity observed in Africans by analyzing the re-sequencing panel with tests of natural selection that are based on the proportions of rare and common polymorphisms (i.e. the allelic spectrum) expected under neutrality. First, we assumed a null hypothesis of neutrality and constant population size (Table 1). While the allelic spectra of non-African populations are consistent with the null hypothesis, Africans show more rare alleles than expected, which is evidenced by negative and significant values (P<0.02) of the Fu-Li's D* and F* statistics. The Tajima's D statistics for the African sample also corresponds to the low fifth-percentile when compared with the 329 genes sequenced in an African-American sample by the Seattle-SNPs initiative (http://pga.gs.washington.edu/summary_stats.html). Based on the contrasting pattern of diversity along *SLC2A4*, we compared the allelic spectra of the regions upstream of exon 7 and downstream of intron 6 and observed that, while Africans show an excess of rare alleles (measured by D*_Fu-Li_ and F*_Fu-Li_) in both regions (data not shown), the presence of 3 singleton and no common variation downstream of intron 6 in the European population is not compatible with the null hypothesis of neutrality (D*_Fu-Li_ = −3.131 and F_Fu-Li_ = −3.134, P<0.05). This comparison was not applied to Asians and Hispanic population because they show no variation downstream of intron 6. These results suggest that under the assumption of constant population size, an observed excess of rare alleles is compatible with a selective sweep or with background selection against deleterious mutations affecting the variation of *SLC2A4* in Africans and Europeans. We also assumed a set of null hypotheses for human populations based on scenarios of demographic expansion. In this case, the excess of rare alleles in Africans is compatible with neutrality under the following scenarios: (a) an exponential growth that started at least 2400 generations (∼60000 years) ago from the 0.001% of the current population size and (b) with a very recent expansion (∼200 generations, ∼5000 years) from the 0.0001% of the current population size. Therefore, *SLC2A4* African allelic spectrum is compatible with an evolutionary history that may involve a combination of population expansion and/or natural selection (selective sweep or background selection).

**Figure 3:**
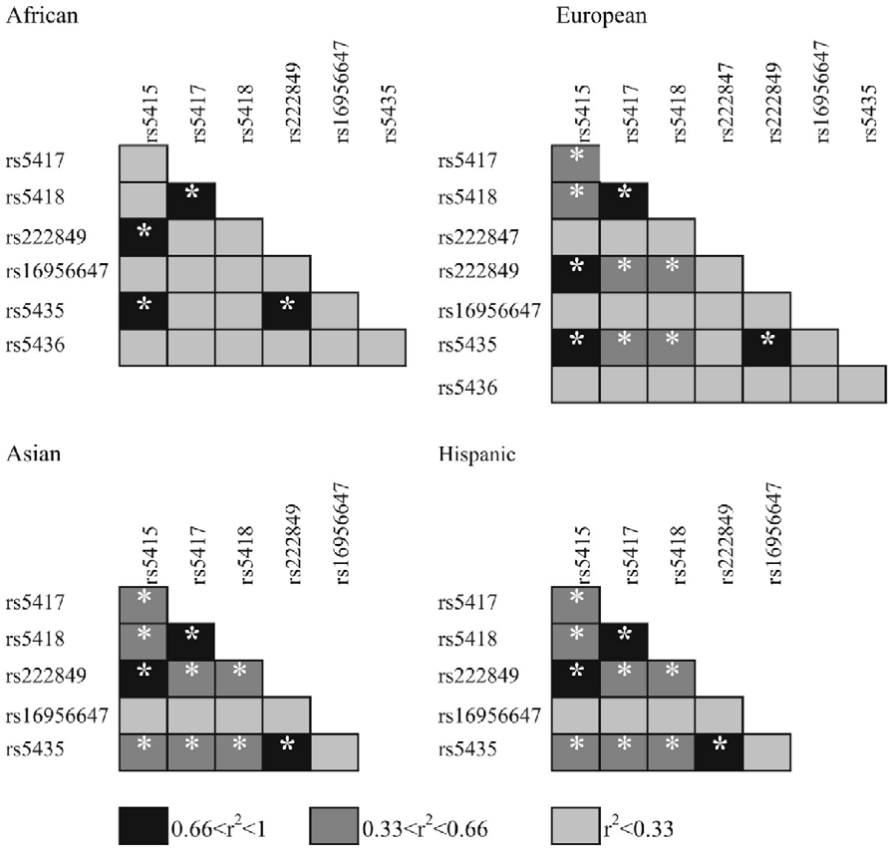
Pairwise linkage disequilibrium in *SLC2A4* in human populations as ascertained in the re-sequencing panel. Significant r^2^ values (LOD >2) are denoted by white asterisks.

For *SLC2A4*, Africans show the highest recombination parameter ρ and the lowest LD, consistent with studies on other genomic regions and with the human evolutionary history ([41], Table 1 and Figure 3), although substantial intragenic LD is shared across human populations. We performed an analysis of long range LD on the genomic region of ∼1 Mb containing *SLC2A4* at its center (see supplementary File S1), to gain information about possible recent events of natural selection. Based on the information from ∼50 SNPs mapped on ∼0.5 Mb at each side of *SLC2A4*, we first verified that there is no statistical evidence of recombination hotspots near *SLC2A4* [32]. Then we determined that this gene is not located within a block of LD in any of the four studied populations. Also, none of the *SLC2A4* common haplotypes is associated with increased measurements of LD, when measured by the Extended-Haplotype-Homozygosity statistic [45]. Thus, we have no evidence of ongoing positive selection associated with this gene.

**Figure 4:**
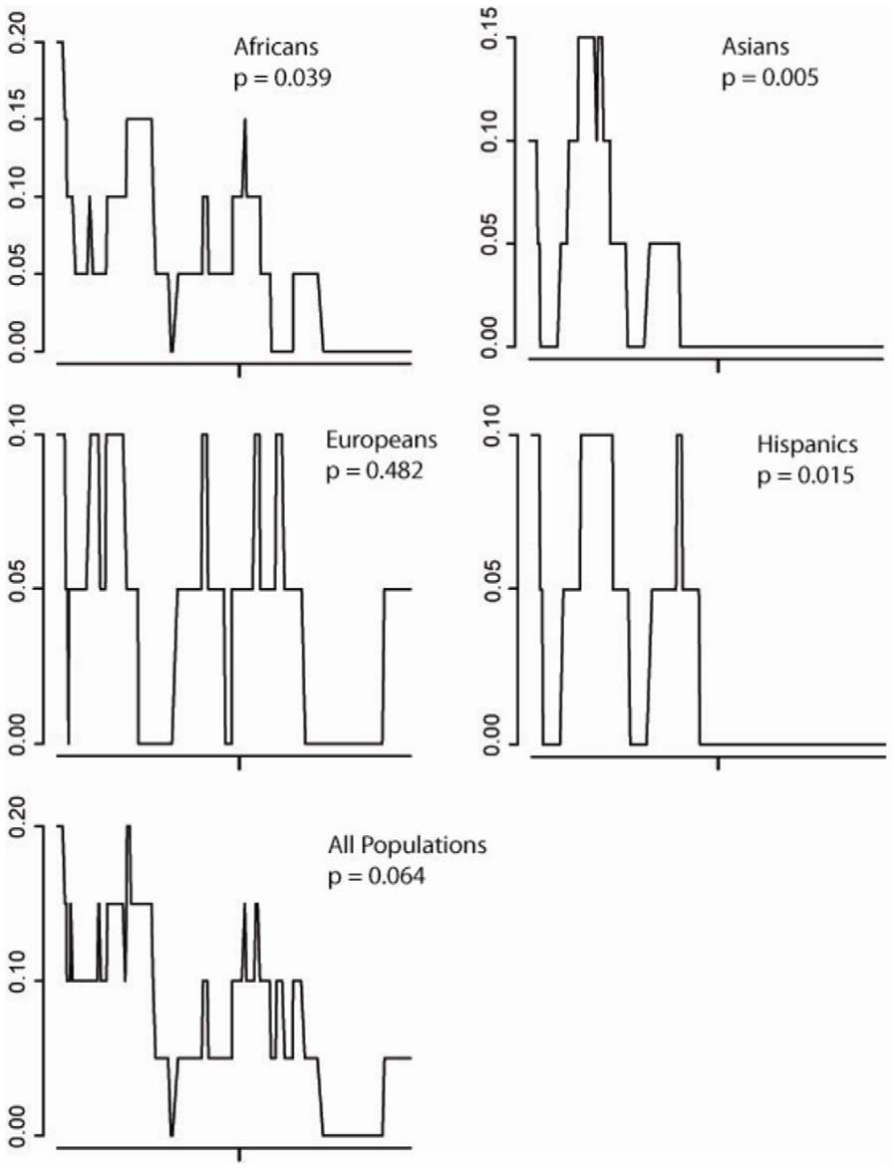
Proportions of polymorphisms to fixed substitutions among humans and rhesus (P/K), calculated by a sliding window approach. Each window includes 20 substitutions. The P value for the Kolmogorov-Smirnoff statistic by McDonald [35] was used to test if the P/K ratio was homogeneous along the gene (see Supplementary File S1 for results using the chimpanzee as outgroup). To be conservative, we evidence the highest P value among those obtained assuming values of recombination parameter r equal to 0, 2, 4 and 6. In the horizontal axes, the vertical tick mark indicates the intron 6- exon 7 boundary. The pattern of significance is the same when *Mus musculus* or *Rattus norvegicus* are used as outgroups. Excluding chimpanzee and rhesus; *M. musculus* and *R. norvegicus* are the mammals most closely related to humans for which *SLC2A4* genomic sequences are available in NCBI databases.

To further assess if the lack of common variants downstream of intron 6 may be due to natural selection at inter-specific level, we applied the Kolmogorov-Smirnov statistic (KS), which belongs to a family of statistics that test if the ratio of polymorphism to divergence along a gene is homogenous, as expected under neutrality [36]. Among these tests, the KS statistic has the highest power to detect patterns in which one end of a gene has high polymorphism and the other end has low polymorphism, as in the case of *SLC2A4*. Moreover, it does not require an arbitrary division of the *SLC2A4* in two parts to be compared (e.g. upstream of exon 7 and downstream of intron 6), a procedure that would be necessary if the classical Hudson-Kreitman-Aguade test (HKA [46]) were applied (but see the supplementary File S1 for results of this classical test). We used two outgroups: chimpanzee (diverged from humans 5–6 millions of years-MY ago) and rhesus monkey (diverged from humans 20–25 MY ago). When we used the chimpanzee as outgroup, we did not reject the null neutral expectation that the ratio of polymorphisms to divergence is homogeneous across *SLC2A4* (supplementary File S1). However, when we used rhesus monkey as outgroup, this pattern changed, and there is significantly less human polymorphisms in Africans, Asians and Latin Americans in the second part of the gene than expected based on the divergence among humans and rhesus (Figure 4). This is even more evident when we consider that all polymorphisms observed downstream of intron 6 are singletons (see also the supplementary File S1 for HKA results). Therefore, if natural selection contributed to reduce the diversity in the second part of *SLC2A4*, this may not be an event restricted to the human evolutionary history, since the comparison with chimpanzee shows that a lower rate of accumulation of substitutions downstream of intron 6 was already evident along the lineages of 5–6 MY that separate humans and chimpanzees. However, divergence downstream of intron 6 accumulated faster in the timeframe between human-rhesus and human-chimpanzee divergences, at rates comparable to the region upstream of exon 7. These results are consistent with an episode of natural selection occurred after the divergence between lineages leading to humans and rhesus (20–25 MY), but predating the divergence between humans and chimpanzee (5–6 MY). Alternatively, the absence of significance observed when the chimpanzee was used as the outgroup may be due to a reduced statistical power determined by few fixed differences between humans and chimpanzees. In this case, natural selection would have not predated the divergence among humans and chimpanzees.

**Figure 5:**
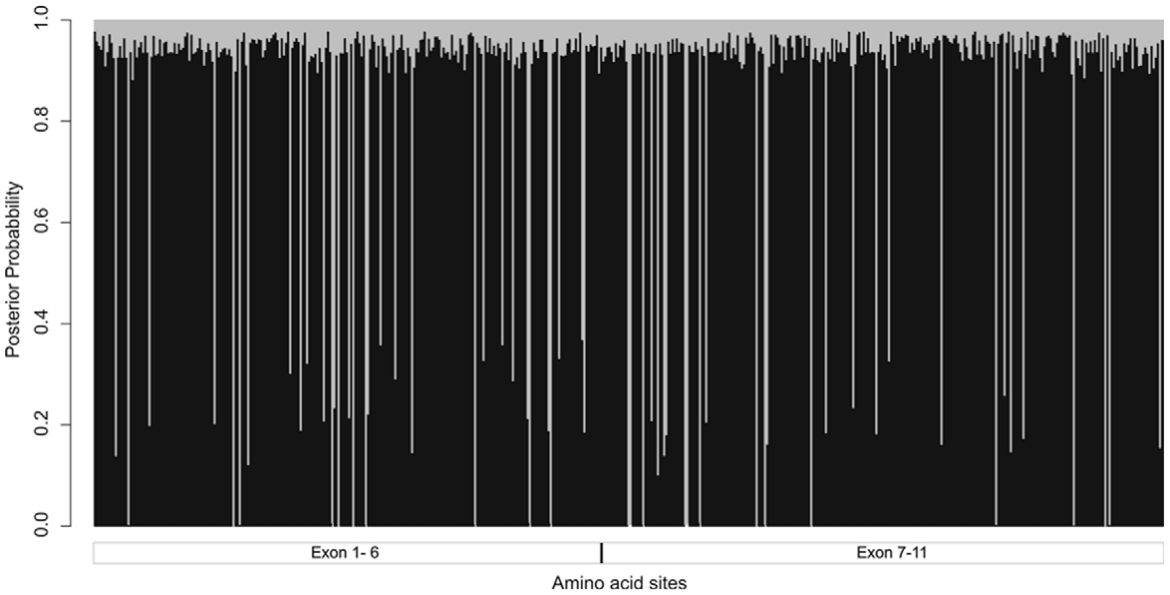
Probability of evolving under strong (ω_s_ = 0.007, in black) or weak (ω_w_ = 0.506, in gray) purifying selection for each of the *SLC2A4* codons (in the horizontal axis).

To determine if the observed pattern of diversity is due to the action of natural selection on *SLC2A4* coding region, we obtained maximum likelihood estimations [37] of the ratios of non-synonymous (dN) to synonymous (dS) substitutions (ω  =  dN/dS) for *SLC2A4* codons under a variety of evolutionary models. The ω parameter is expected to be 1 under neutrality, <1 (dN < dS) under purifying selection and >1 (dN > dS) under positive selection. The best fit of our data is obtained for models that (see the supplementary File S1 for detailed results): (1) allow for values of ω≤1 to vary across *SLC2A4* coding region, (2) do not show strong evidence of relaxation of purifying selection along the primate lineages and, (3) do not show evidence of positive selection. In particular, the discrete Model 3 of Yang [37], that allow for K = 2 different classes of ω (without restrictions for the value of this parameter) best fit the data, and suggests that ∼85% of *SLC2A4* codons evolved under strong purifying selection (ω≈0.007) and ∼15% under a weaker purifying selection (ω≈0.506, Figure 5). There is no association among the distribution of these two classes of codons and their location in the transmembrane domains of GLUT4. Also, codons that evolved under strong purified selection are not associated (Fisher exact test P = 0.41) with the region encompassing exons 7–11, where no common polymorphisms are present in humans and a reduced rate of accumulation of substitutions is observed along the chimpanzee-human genomic lineage. This result suggests that our results for the Kolmogorov-Smirnov test, possibly attributed to the action of natural selection, do not depend on variation in the *SLC2A4* coding region.

## Discussion

Considering the evolutionary timeframe of mammals, we observed no evidence of positive natural selection for the *SLC2A4* coding sequence, although inferences about ω using the Yang [37] approach has sufficient power for a protein with more than 500 codons, such as GLUT4 [47], [48]. While most codons (∼85%) are under strong purifying selection, for sixty of them (15%) purifying selection was weaker. In fact, codons of the latter category present non-synonymous substitutions (19 of them more than one at the same codon) along the mammal phylogeny. Classifying *SLC2A4* codons in two classes of purifying selection is a simplification, but we think this is a reasonable assignment that derives from the evolutionary model that best fit our data (Model 3 of Yang [37], supplementary File S1). In any case, this simplification allowed us to verify that these classes of codons are not associated with portions of *SLC2A4* upstream of exon 7 or downstream of intron 6. Therefore, the pattern of substitution across the phylogeny of mammals coding region does not explain the lack of common variation in humans nor the lower divergence along the human-chimpanzee lineages for the second part of the gene.

We observed that when we used the rhesus monkey (that diverged from humans 20–25 MY ago) as outgroup and applied the Kolmogorov-Smirnov neutrality test, we do not observe along the human-rhesus lineages the paucity of variation downstream of intron 6 that is observed for human polymorphisms. We interpret this result as evidence that natural selection reduced the variability downstream of *SLC2A4* intron 6 during the last 25 MY, and the current pattern of diversity observed in modern humans reflects this event. However, an alternative explanation is that comparisons with the chimpanzee - an evolutionarily closed outlier; have less statistical power than comparisons with the rhesus monkey and therefore, our data may be also compatible with a more recent action of natural selection, though not recent enough to be detected using neutrality tests based on linkage disequilibrium [33]. Because we did not observe relevant changes in ω along the primate phylogeny of *SLC2A4* coding sequence, we hypothesize that natural selection acted on a non-coding region of *SLC2A4*. In fact, only neutrality tests such as the KS statistic, which application is not limited to coding regions, are able to capture a pattern like this. Two kinds of selection may reduce genetic diversity: background purifying selection and a selective sweep leading to a hitchhiking event [49]. However, it is unlikely that background purifying selection started to act on a large non-coding region only at a certain point during the last 20–25 MY, after the divergence of humans and rhesus lineages. Instead, a selective sweep is consistent with the lack of variation along a genomic region (such as the second part of *SLC2A4*), with the low nucleotide diversity observed in African populations and with the excess of rare alleles and negative values of the Tajima statistics for the region downstream of intron 6 in Africans and Europeans (although this may be due in part to the demographic history of these populations as suggested by coalescent simulations). What is not inconsistent with a selective sweep scenario, but makes it less likely, is the fact that the observed lack of variation is mainly restricted to the region downstream of intron 6, and we did not find evidence for the existence of a recombination hotspot within the *SLC2A4* locus that prevents the propagation of the signature of natural selection along a larger genomic region. In favor of consistency with a selective sweep scenario, we may also mention that *SLC2A4* is within a genomic region where LD is in general low (supplementary File S1), and therefore, the signature of natural selection determined by a selective sweep would be necessarily restricted to a small region. If a complete selective sweep occurred during the last 20–25 MY along the rhesus-human lineage, this may be compatible with a “transpecies” version of the “thrifty” genotype hypothesis (see Introduction of [8]). In this hypothetical scenario, we may not see association between diabetes susceptibility and *SLC2A4* variants [50] because a selective sweep lead to the existence of a small genomic region with no common variants, and the fixed haplotype may be “thrifty”. By examining the pattern of long-range LD, we did not find evidence of an ongoing selective sweep within a temporal frame of ∼25000 years (the timescale at which a selective sweep left a signature in the pattern of LD, [33]). In fact, none of the common *SLC2A4* haplotypes (defined by SNPs upstream of exon 7) is associated to a large surrounding region of LD - a pattern expected under a recent selective sweep.

Because population samples included in this study (as in most human population genetics studies) are not optimal for the population genetics inferences to be addressed, it is important to consider the limitations of our results. By genotyping five SNPs in an additional worldwide samples from the HGDP-CEPH Panel, we found a haplotype structure that was consistent with that observed in the re-sequencing panel. Although African and Asian/Oceanian samples include individuals with diverse origin and therefore, are structured, we would not expect the paucity of variation observed downstream of intron 6, or the excess of rare alleles in the African sample to be an artifact of our sample composition. Instead, the population structure observed in the African and Asian samples is expected to generate a deficit of rare alleles (and an excess of common alleles), and therefore, our results reporting an excess of rare alleles (or the lack of common variants) are conservative in light of our sampling strategy [25].

In conclusion, after performing extensive sequencing of *SLC2A4*, we determined that it has a peculiar pattern of genetic variation, with the first part of the gene showing common and rare variants in a fashion compatible with neutral evolution. However the second part of the gene shows no common variants as well as a pattern of diversity that is not compatible with neutrality, but compatible with an event of natural selection that reduced the level of substitution in this region during the last 20–25 MY. Although the natural selection scenario is compatible with the observed data, we recommend caution since claims of natural selection should require replication on larger samples to be accepted, and if possible, understanding of its biological/functional basis.

## Supporting Information

File S1 (3.13 MB DOC)
