# Supplementary material for: Diversity in the Glucose Transporter-4 Gene (SLC2A4) in Humans Reflects the Action of Natural Selection along the Old-World Primates Evolution
Source: PLoS One. 2010 Mar 23;5(3):e9827. doi: 10.1371/journal.pone.0009827 (PMC2843742; doi:10.1371/journal.pone.0009827)
Supplement: File S1 — (3.13 MB DOC) [file pone.0009827.s001.doc]

**SUPPLEMENTARY MATERIAL**

**Diversity in the Glucose Transporter-4 gene (*SLC2A4*) in Humans Reflects the Action of Natural Selection along the Old-World Primates Evolution**

Eduardo Tarazona-Santos ^1,2,*^, Cristina Fabbri ^3,1,*^, Meredith Yeager ^4^, Wagner CS Magalhaes ^1,2^, Laurie Burdett ^4^, Andrew Crenshaw ^4^, Davide Pettener ^3^, Stephen J Chanock ^1^

^1^ Laboratory of Translational Genomics of the Division of Cancer Epidemiology and Genetics, National Cancer Institute (NCI), National Institutes of Health (NIH), Gaithersburg, Maryland, USA.

^2^ Departamento de Biologia Geral, Instituto de Ciências Biológicas, Universidade Federal de Minas Gerais. Belo Horizonte, Minas Gerais, Brazil.

^3^ Dipartimento di Biologia Evoluzionistica Sperimentale, Università di Bologna, Bologna, Italy.

^4^ Intramural Research Support Program, SAIC Frederick, NCI-FCRDC, Frederick, Maryland, USA and Core Genotype Facility, NCI, NIH, Gaithersburg, Maryland, USA.

* These authors equally contributed to this paper.

**Technical specifications of the *Material and Methods* section.**

We inferred haplotypes and inferred the recombination parameter ρ using the software Phase v.2.1.1., using 10.000 iterations, thinning intervals of 100 and burn in of 1000.

For coalescent simulations of exponential growth scenarios, we used the software ms, developed by Richard Hudson, conditioned the simulations (5000 repetitions) on the *θ* estimator π, and simulated models of exponential growth from 0.0001, 0.001, 0.01, 0.1 and 1.0% of the current population size that started 200, 400, 800,… 4000 generations ago. As π is an estimator of *θ = 4N_e_μ* that reflects the effective population size prior to the population expansion rather than current *θ* values (Tajima 1989), we conditioned simulations of X-fold population expansions based on estimators of *θ* equal to Xπ. As required by the ms software, time was measured in units of 4N_0_ = Xπ/μ.

For phase inferences for the long range LD analysis using 104 Affymetrix SNPs, we used the fastPHASE software using the following parameters: 10 clusters, 20 random starts and 25 iterations for the EM algorithm. To infer recombination hotspots we used the software SequenceLDhot, using the following parameters: 5000 runs, a minimum of 100 iterations per hotspot, capturing information from windows of 7 consecutive SNPs (as recommended by the author), assuming 2kb as expected hotspot length and testing for a hotspot every 1 kb.

**Summary of the results from the SNPs-panel**

| Results of the SNPs panel may be summarized as follow: (1) Haplotypes inferred from these 5 SNPs and their frequencies are consistent with the haplotypes inferred from 8 common SNPs of the re-sequencing panel (Supplementary Table 1). (2) Considering haplotype diversity, the African population is the most diverse, followed by the Asian one (Supplementary Table 1). (3) When the thirteen worldwide populations are considered, the F_ST_ is low (5.71%, P < 0.01); and when these populations are pooled in four continental groups (Europeans, Africans, Asians and Native Americans) the among-groups component of the total genetic variance (F_CT_) is 4.9% (P < 0.01). Also for the SNPs-panel, most of the worldwide between-population genetic variance is due to comparisons among African and non-African populations (pairwise F_CT_ = 8.3%, P = 0.001, see Supplementary Table 2 and Figure S1).  **Supplementary Table 1.** *SLC2A4* inferred haplotypes and their absolute frequencies in the SNPs-panel populations. | | | | | | | | | | | |
| --- | --- | --- | --- | --- | --- | --- | --- | --- | --- | --- | --- |
| Haplotypes | rs5417 | rs5418 | rs16956647 | rs5435 | rs5436 |  | African | Native American | European | Asian | World |
|  |  |  |  |  |  |  |  |  |  |  |  |
| A1-5-6 | **C** | **G** | **C** | **C** | **C** |  | 73 | 7 | 11 | 8 | 99 |
| A2 | **.** | **.** | **.** | **.** | **T** |  | 14 | 1 |  |  | 15 |
| A3 | **.** | **.** | **T** | **.** | **.** |  | 7 | 7 | 1 | 23 | 38 |
| A4-7 | **.** | **.** |  | **T** | **.** |  | 18 | 31 | 36 | 57 | 142 |
| A8 | **.** | **.** | **.** | **T** | **T** |  | 1 |  |  |  | 1 |
|  |  |  |  |  |  |  |  |  |  |  |  |
| AB1 | **.** | **A** | **.** | **.** | **.** |  |  |  | 2 |  | 2 |
| AB2 | **A** | **.** | **.** | **.** | **.** |  | 3 |  | 1 | 3 | 7 |
| AB3 | **A** | **.** | **.** | **T** | **.** |  |  | 1 | 1 |  | 2 |
| AB4 | **A** | **.** | **T** | **.** | **.** |  |  | 1 |  |  | 1 |
|  |  |  |  |  |  |  |  |  |  |  |  |
| B1-3 | **A** | **A** | **.** | **.** | **.** |  | 36 | 50 | 77 | 79 | 242 |
| B2-4 | **A** | **A** | **.** | **T** | **.** |  |  |  | 3 | 8 | 11 |
|  |  |  |  |  |  |  |  |  |  |  |  |
| Number of chromosomes | | | | | | | 152 | 98 | 132 | 178 | 560 |
| Number of common haplotypes | | | | | | | 7 | 7 | 8 | 6 | 11 |
| Number of common polymorphic sites | | | | | | | 5 | 5 | 4 | 4 | 5 |
| Haplotype diversity | | | | | | | 0.693 | 0.636 | 0.582 | 0.683 | 0.713 |
| S.D. of haplotype diversity | | | | | | | 0.028 | 0.034 | 0.036 | 0.021 | 0.012 |
|  |  |  |  |  |  |  |  |  |  |  |  |

**Supplementary Table 2.** Matrix of pairwise F_ST_ among continental groups of populations genotyped for the SNPs-panel (rs5418, rs16956647, rs5435, rs5436, and rs5417).

|  | African | Nat. Amer. | European |
| --- | --- | --- | --- |
| African |  |  |  |
| Nat. Amer. | 0.082* |  |  |
| European | 0.084* | 0.000 |  |
| Asian | 0.115* | 0.005 | 0.029* |
| * P < 0.02 |  |  |  |

**Figure S1.** Analysis of Molecular Variance results and bi-dimensional Non-Metric Multidimensional Scaling of the matrix of pairwise F_ST_ among the 13 populations of the SNP-panel.

**Figure S2.** Proportions of polymorphisms to fixed substitutions among humans and chimpanzee (P/K), calculated by a sliding window approach. Each window includes 20 substitutions. The P value for the Kolmogorov-Smirnoff statistic by McDonald (1998) was used to test if the P/K ratio was homogeneous along the gene. To be conservative, we evidence the highest P value obtained assuming values of recombination parameter ρ equal to 0, 2, 4 and 6.


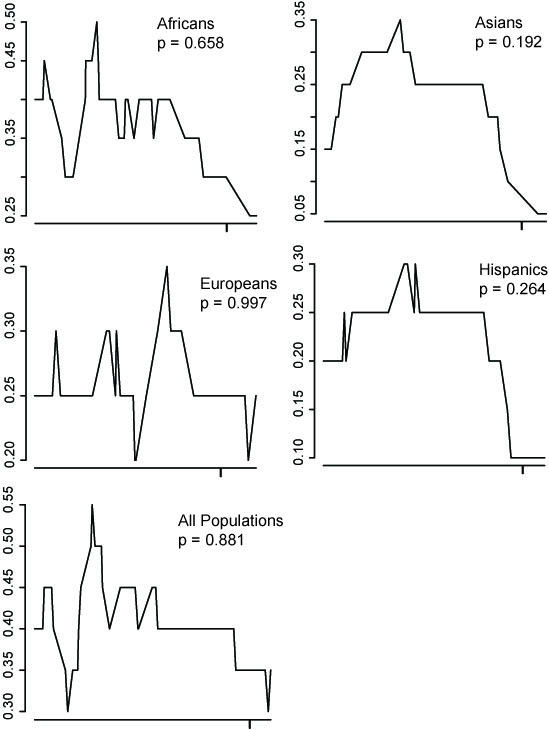


**Figure S3.** Pairwise linkage disequilibrium in the four population of the re-sequencing panel along 0.5Mb at each side of the *SLC2A4* locus (within the circle). LD is measured using the r^2^ statistic and 56 and 48 SNPs at the left and right sides of *SLC2A4* respectively, plus the 8 common *SLC2A4* SNPs.

Affymetrix SNPs used in the long range LD analysis, and their positions (based on the NCBI human genome build 36.3) in parenthesis:

rs1509122 (6634190), rs2309624 (6634762), rs7220599 (6634969), rs17804635 (6644709), rs17804647 (6644795), rs2136282 (6644997), rs7224971 (6654723), rs4796560 (6656402), rs12103615 (6661906), rs12103645 (6661940), rs12103647 (6662051), rs4796355 (6662311), rs4796356 (6662514), rs9914907 (6666473), rs9899579 (6666836), rs9894204 (6667096), rs9894214 (6667109), rs9895170 (6667515), rs9901847 (6670274), rs9903322 (6677426), rs16956324 (6738539), rs573836 (6739102), rs2271315 (6743013), rs311758 (6743364), rs311752 (6784050), rs9630721 (6791092), rs9913738 (6811343), rs311738 (6821360), rs311740 (6822833), rs6502997 (6828482), rs312471 (6843990), rs11571340 (6848943), rs312462 (6854376), rs11571353 (6855064), rs11078662 (6860981), rs17732290 (6873401), rs1044930 (6874312), rs368900 (6874606), rs9675089 (6901871), rs12950215 (6901941), rs193399 (6902996), rs4796366 (6903235), rs732428 (6909787), rs9892632 (6913074), rs17732397 (6915663), rs7218942 (6915690), rs9900839 (6932166), rs12951306 (6961231), rs2062737 (6972362), rs7207110 (6983567), rs220992 (6990512), rs4464125 (6991999), rs4558460 (6992193), rs314253 (7032374), rs429807 (7054800), rs2654185 (7124086), rs17805277 (7142477), rs17732541 (7144074), rs8076120 (7157487), rs6503013 (7158208), rs2292067 (7192872), rs7214863 (7214801), rs7219773 (7223868), rs2269762 (7226555), rs6503018 (7232831), rs2075759 (7233409), rs2075760 (7233450), rs8075287 (7235795), rs4796305 (7276779), rs4796418 (7298724), rs4796420 (7320341), rs4796421 (7320374), rs3867594 (7321405), rs4491591 (7326779), rs4627421 (7335005), rs9646411 (7339633), rs12937610 (7346661), rs11658168 (7346858), rs7224988 (7350605), rs7215922 (7350936), rs9889368 (7352936), rs2228133 (7353127), rs2269459 (7353762), rs6608 (7405137), rs8068222 (7409412), rs11078697 (7409953), rs10438740 (7413608), rs4602096 (7414181), rs9898876 (7467687), ldgh2 (7471229), rs1641525 (7489936), rs1641523 (7490406), rs1050540 (7501467), rs1050541 (7501560), rs12951053 (7518132), rs2909430 (7519370), rs12602273 (7523738), rs2543540 (7572396), rs16956936 (7574417), rs839721 (7580474), rs12453814 (7606018), rs2871358 (7616198), rs4791759 (7618652), rs6503060 (7628907)

**Supplementary** **Table 3.** Hudson-Kreitman-Aguade neutrality test comparing the two parts of the gene, using *Pan troglodites* and *Macaca mulatta* as outgroups.

|  | *Pan troglodites* | | |  | *Macaca mulatta^1^* | | |
| --- | --- | --- | --- | --- | --- | --- | --- |
|  | 5'-region: upstream exon 7 (3692 bp) | 3'region: downstream intron 6 (2431 bp) |  |  | 5'-region: upstream exon 7 (2815 bp) | 3'region: downstream intron 6 (2590 bp) |  |
| *Africans* |  |  |  |  |  |  |  |
| Polymorphisms | 18 | 2 | Chi-sq = 0.73 |  | 14 | 2 | **Chi-sq = 4.22** |
| Divergence | 32.42 | 8.04 | P = 0.390 |  | 139.69 | 117.04 | **P = 0.039** |
| *Europeans* |  |  |  |  |  |  |  |
| Polymorphisms | 10 | 3 | Chi-sq = 0.06 |  | 8 | 3 | Chi-sq = 1.31 |
| Divergence | 33.28 | 8.05 | P = 0.799 |  | 139.70 | 117.05 | P = 0.309 |
| *Asians* |  |  |  |  |  |  |  |
| Polymorphisms | 8 | 0 | Chi-sq = 1.64 |  | 7 | 0 | **Chi-sq = 4.52** |
| Divergence | 33.20 | 8.00 | P = 0.200 |  | 139.66 | 117.00 | **P = 0.034** |
| *Hispanics* |  |  |  |  |  |  |  |
| Polymorphisms | 9 | 0 | Chi-sq = 1.82 |  | 7 | 0 | **Chi-sq = 4.48** |
| Divergence | 33.09 | 8.00 | P = 0.178 |  | 139.65 | 117.00 | **P = 0.034** |
| *All populations* |  |  |  |  |  |  |  |
| Polymorphisms | 20 | 5 | Chi-sq = 0.002 |  | 16 | 5 | Chi-sq = 2.60 |
| Divergence | 33.01 | 8.02 | P = 0.968 |  | 139.68 | 117.02 | P = 0.107 |
|  |  |  |  |  |  |  |  |

^1^The pattern of significance is the same when *Macaca mulatta, Mus musculus* or *Rattus norvegicus* are used as outgroups. Excluding chimpanzee and rhesus; *M. musculus* and *R. norvegicus* are the mammals most closely related to humans for which *SLC2A4* genomic sequences are available on the NCBI databases.

**Supplementary** **Table 4.** Evolutionary analysis of *SCL2A4* using the maximum likelihood approach of Yang (2007a), as implemented in the software PAML.

| Model | Description | Number of Parameters | Log likelihood | Estimated parameters |
| --- | --- | --- | --- | --- |
| M0 | One ω ratio for the entire sequence and phylogeny | 1 | -4261.73 | ω = 0.072 |
| *Site specific models* | |  |  |  |
| M1-neutral | Two classes of codons: Fraction p_0_ of codons with ω_0_<1 and p_1_=(1-p_0_) with ω_1_=1 | 2 | -4230.23 | p_0_ = 0.923, ω_0_ = 0.024 |
| M2-positive selection | As M1 with one additional class of codons (p_2_) with ω_2_>1 | 4 | -4230.23 | p_0_ = 0.923, p_1_ = 0.076,  p_2_  = 0.000, ω_0_ = 0.024 |
| M3-discrete general | K=3 classes of sites: each fraction of p_k_ of sites with its  ω _k_ | 5 | -4227.50 | p_0_ = 0.082, p_1_= 0.768, p_2_ = 0.150, ω_0_ = 0.007, ω_1_ = 0.007, ω_2_ = 0.506 |
| M7- beta neutral | ω values (within 0-1 interval) fit a beta distribution with parameters p and q | 2 | -4227.61 | p = 0.079, q = 0.843, ω_avg_ = 0.083 |
| M8-beta and positive selection | Fraction p_0_ of codons as M7, with an additional class of p_1_ codons with ω > 1 | 4 | -4227.31 | p_0_ = 0.998, p = 0.085, q = 0.926, p_1_ = 0.001, ω_1_ = 9.311 |
| *Branch-site models* | |  |  |  |
| Model A – neutral | Portions p_0_ and (p_1_+p_3_) of codons have ω_0_ < 1 and ω_1_ = 1 respectively across the entire phylogeny. A portion p_2_ of codons have ω < 1 in the background of phylogeny and ω = 1 in the foreground of phylogeny (primates) | 3 | -4230.33 | p_0_ = 0.923, p_1_ = 0.077,  ( p_2_ + p_3_ = 0), ω_0_ = 0.025 |
| Model A - positive selection | As Model A but instead of neutral, portions p_2_ and p_3_ of codons have ω > 1 in the foreground of the phylogeny (primates) | 4 | -4230.33 | p_0_ = 0.923, p_1_ = 0.077,  (p_2_ + p_3_ = 0), ω_0_ = 0.024 |
| *Clade models* | | | | |
| Model C | Fraction p_0_ and p_1_ of codons evolve with ω _0_ < 1 and ω _1_=1 across the entire phylogeny, and p_2_ under ω _2_ on the background of the phylogeny and ω _3_ on the foreground of the phylogeny (primates) | 5 | -4226.00 | p_0_ = 0.858, p_1_ = 0.00, p_2_ = 0.15, ω _0_ = 0.010, ω _2_ = 0.577 , ω _3_ = 0.229 |

Comparison of models by a Likelihood Ration Test:

M0 vs. M3, 2ΔL = 68.46, df = 4, P < 0.0001.

M1 vs. M2, 2ΔL = 0.00, df = 2, P = 1.00.

M7 vs. M8, 2ΔL = 0.60, df = 2, P = 0.74.

Model A neutral vs. Model A with positive selection, 2ΔL = 0.00, df = 1, P = 1.00.

M1 vs. Model C, 2ΔL = 8.46, df = 3, P = 0.037.
